# UAB/SNMMI Nuclear Medicine Therapy Intensive: Advancing Competence and Innovation in Radiopharmaceutical Therapy

**DOI:** 10.2967/jnmt.125.271222

**Published:** 2025-12

**Authors:** Krystle W. Glasgow, Amy B. Brady

**Affiliations:** Department of Clinical and Diagnostic Sciences, University of Alabama at Birmingham, Birmingham, Alabama

**Keywords:** radiopharmaceutical therapy, radiation safety, therapy, intensive

## Abstract

The rapid expansion of radiopharmaceutical therapy, fueled by new radiopharmaceuticals approved by the Food and Drug Administration and novel theranostic approaches, has created an urgent demand for advanced training beyond foundational nuclear medicine education. To address this gap, the University of Alabama at Birmingham and the Society of Nuclear Medicine and Molecular Imaging launched the Nuclear Medicine Therapy Intensive in 2024. Designed for practicing nuclear medicine technologists and nuclear medicine advanced associates, this program integrates didactic instruction, simulation-based learning, interprofessional collaboration, and innovative capstone experiences (e.g., a nuclear medicine therapy escape room). Participants engage in activities that strengthen their knowledge in radiation safety, dosimetry, patient eligibility, communication, and clinical trial literacy while applying skills in hands-on therapy simulations. Preassessment and postassessment results from 2024 and 2025 cohorts demonstrated marked gains in knowledge and confidence, with most graduates directly working with theranostics services at their institutions. The weeklong intensive serves as a model for preparing professionals to deliver radiopharmaceutical therapies safely and effectively and with patient-centered excellence.

## Visual Abstract

**Figure:**
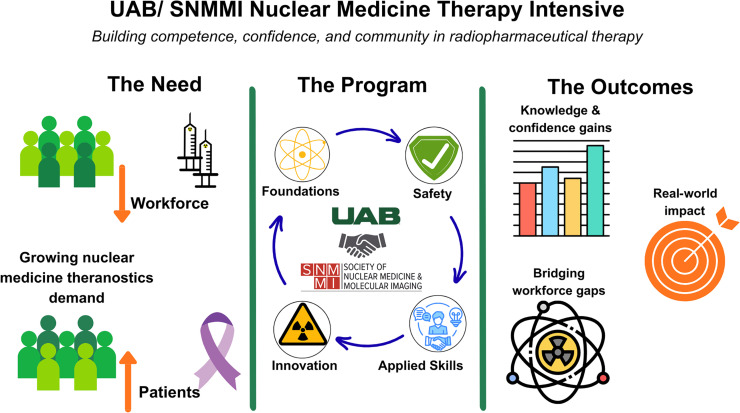

## MEETING AN URGENT WORKFORCE AND COMPETENCY NEED

Radiopharmaceutical therapy (RPT) is undergoing unprecedented expansion, driven by the approval of new radiopharmaceuticals by the Food and Drug Administration, the rise of novel theranostic approaches, and increasing integration of cancer treatment pathways. This rapid growth has created a pressing need for qualified professionals who can deliver these therapies safely, effectively, and in accordance with complex regulatory requirements. Delivering RPT demands more than foundational training. It demands advanced, targeted education in dosimetry, patient-specific treatment planning, radiation safety, and interprofessional collaboration (1).

The need is compounded by workforce pressures. A 2023 editorial in the *Journal of Nuclear Medicine* described worsening workforce shortages in the United States due to limited training pipelines (2). A national consensus report highlighted projected annual vacancies across radiologic professions including nuclear medicine technologists (NMTs) (3). Globally, surveys show that many countries are ill-equipped with trained personnel and infrastructure to meet current and growing demand (1,4).

Introduced in 2024, the University of Alabama at Birmingham (UAB)/Society of Nuclear Medicine and Molecular Imaging (SNMMI) Nuclear Medicine Therapy Intensive was developed to address this need. Designed for board-certified NMTs and nuclear medicine advanced associates (NMAAs) already in practice, the weeklong program provides advanced, practice-focused education that prepares participants to lead in this evolving specialty.

### Mission and Educational Goals

The mission of the UAB/SNMMI Nuclear Medicine Therapy Intensive is to provide qualified nuclear medicine professionals with advanced knowledge and practical skills necessary for excellence in RPT.

The program’s learning outcomes reflect the competencies needed for safe and effective practice:

1. Identify principles of RPT.
2. Master radiation safety practices.
3. Evaluate patient eligibility, preparation, and communication strategies.
4. Perform radiopharmaceutical preparation and administration.
5. Monitor patient response and follow-up care.
6. Collaborate as part of an interprofessional team.
7. Demonstrate competence in practical skills through simulation and assessment.

### Curricular Themes

The curriculum integrates theoretic knowledge, applied skills, and interactive learning across 6 major themes (Table 1).

**TABLE 1.** Mapping of UAB/SNMMI Nuclear Medicine Therapy Intensive Learning Themes to Program Activities

Curriculum theme	Representative activities
Foundations in RPT	Lecture: Introduction to theranostics Lecture: Next-generation targets for radiopharmaceuticals Overview of radiopharmaceutical procedures Tour: UAB Advanced Imaging Facility and cyclotron
Radiation safety and compliance	Lecture: Radiation safety/regulations with RPTs Lab: Radiation safety demonstration Review of NRC/state protocols and contamination control procedures
Patient-centered communication and eligibility	Consultation Communication Lab Interprofessional floor exercise NMAA panel discussion Case study reviews on patient selection and preparation
Simulation and applied skills	Therapy simulation lab: mock patient setup, dose calculation, and administration workflow Division of Molecular Imaging and Therapeutics tour of the therapy suite Dosimetry software virtual lab and demonstrations Hands-on protocol review and therapy project work
Clinical trials and research literacy	Lecture: Approved therapies and clinical trials Lecture: Clinical trials: nuts and bolts Project: Design of technologist study guide for therapy workflows
Innovative application—nuclear medicine therapy escape room	Capstone nuclear medicine therapy escape room integrating dose calculation, radiopharmaceutical selection, workflow problem-solving, and safety decision-making in a timed, team-based format
Program integration and assessment	Pretests and posttests Team therapy project presentations Therapy Hunger Games challenge

NRC = Nuclear Regulatory Commission.

## FOUNDATIONS IN RPT

**FIGURE 1.**
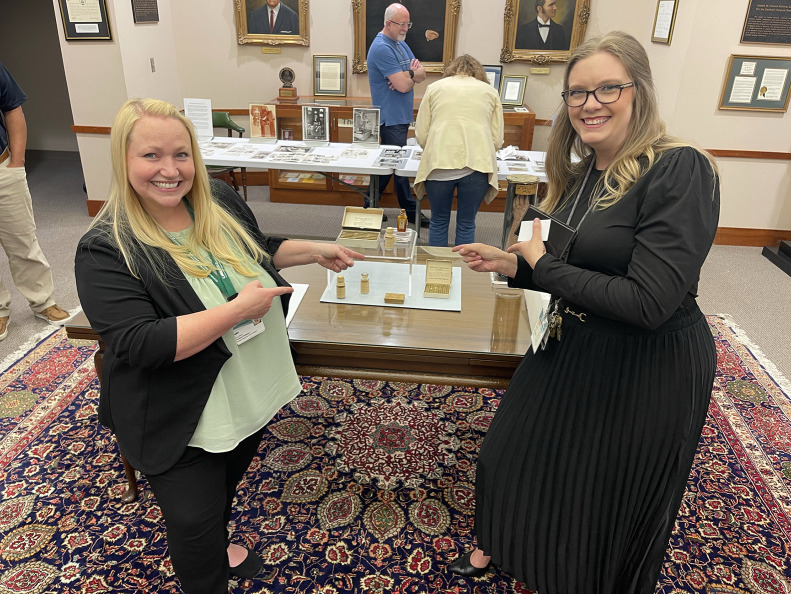
Participants tour the Alabama Museum of the Health Sciences.

The curriculum began by grounding participants in core principles of theranostics, the molecular basis of targeted therapy, and emerging radiopharmaceutical development. The intensive brought in expert faculty to connect scientific advances to patient outcomes, emphasizing the importance of individualized therapy selection. Participants concluded the session with a guided tour of the Alabama Museum of the Health Sciences, providing historical context on the progress that shaped modern RPT (Fig. 1).

## RADIATION SAFETY AND COMPLIANCE

**FIGURE 2.**
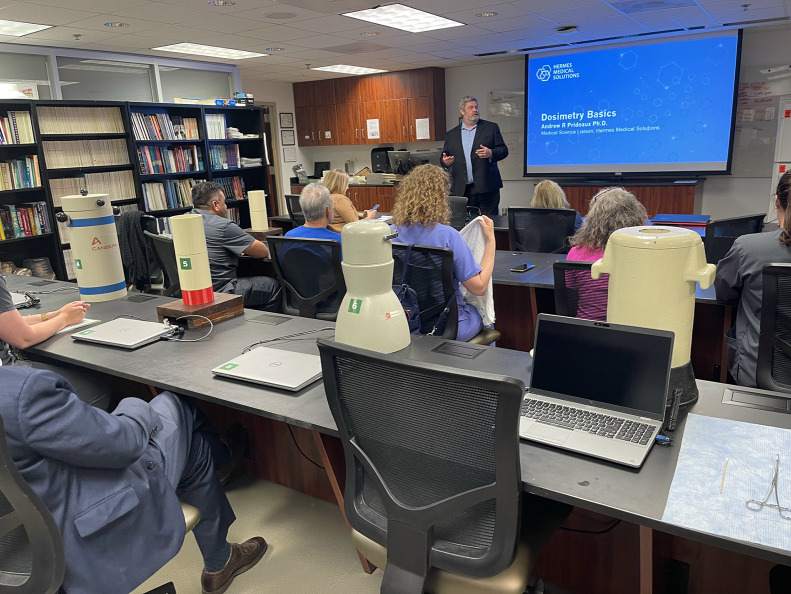
Participants learn about radiation safety and dosimetry in the UAB Norman E. Bolus Nuclear Medicine and Health Physics Laboratory.

Participants strengthened their understanding of safety regulations, shielding, contamination control, and posttherapy precautions through a combination of lectures, labs, and protocol reviews (Fig. 2). The focus was on translating regulatory requirements into daily best practices that provide protection for patients, staff, and the general public.

## PATIENT-CENTERED COMMUNICATION AND ELIGIBILITY

Interactive labs, role-playing, and case reviews prepared participants to navigate patient eligibility assessment, informed consent, and therapeutic counseling. Real-world insights from practicing NMAAs illustrated the nuances of patient communication in high-stakes care settings.

## SIMULATION AND APPLIED SKILLS

**FIGURE 3.**
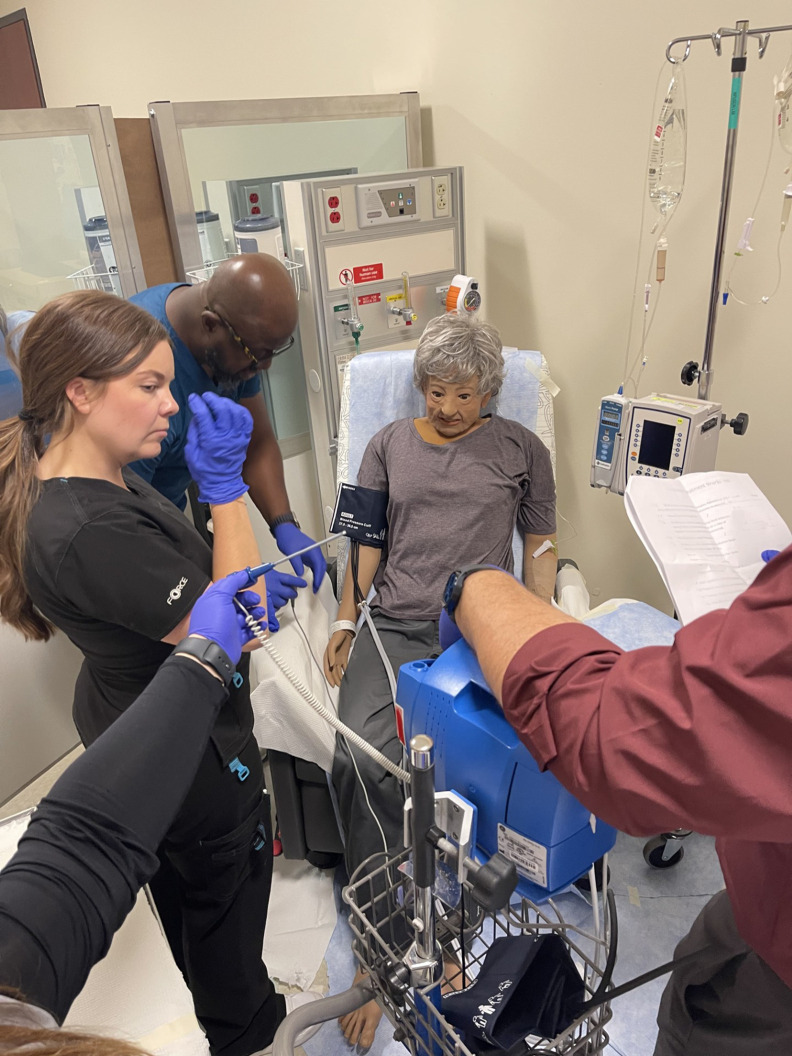
Participants work through guided simulations and practice preparing patients for RPT.

**FIGURE 4.**
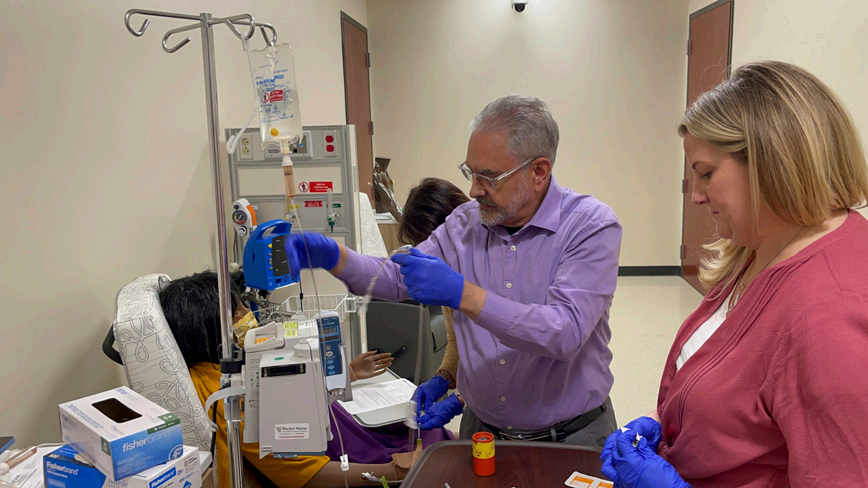
Participants practice administering RPTs to patients through simulation.

From therapy suite tours to hands-on simulation labs, participants engaged in a comprehensive exploration of the RPT workflow. Through guided simulations, they practiced preparing and administering therapeutic radiopharmaceuticals, reinforcing procedural competence and safety principles (Figs. 3 and 4). Virtual dosimetry labs allowed exploration of imaging-based treatment planning, reinforcing the connection between diagnostic imaging and the delivery of RPT.

## CLINICAL TRIALS AND RESEARCH LITERACY

**FIGURE 5.**
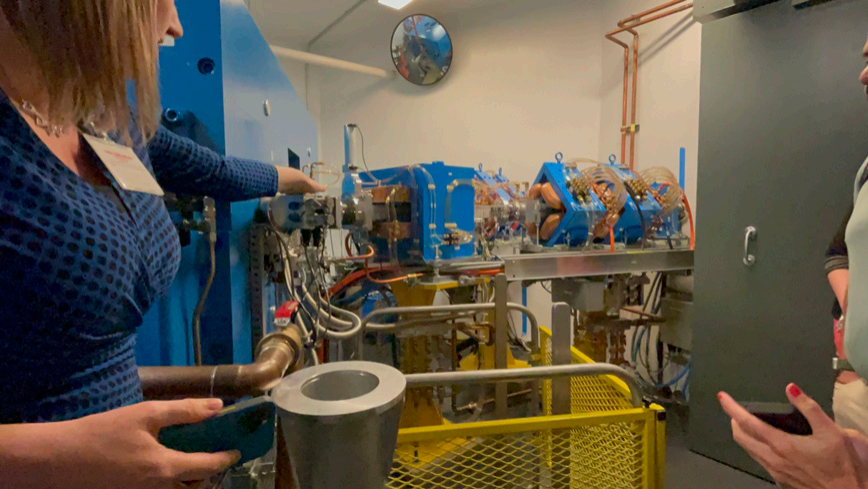
Participants tour the UAB Advanced Imaging Facility to learn about cyclotron operations and development of novel radiopharmaceuticals.

Through direct engagement with clinical trial experts, participants learned how research informs therapy protocols and how technologists can contribute to evidence generation and innovation (Fig. 5).

### Engagement with Industry

A defining feature of the intensive was its close partnership with industry leaders. Multiple vendors and corporate partners were actively involved throughout the weeklong event, offering lectures on emerging technologies, practical demonstrations, and hands-on training with software. This collaboration ensured that participants not only received instruction of the theory behind theranostics but also gained direct exposure to the tools, techniques, and workflows shaping the future of RPT.

**FIGURE 6.**
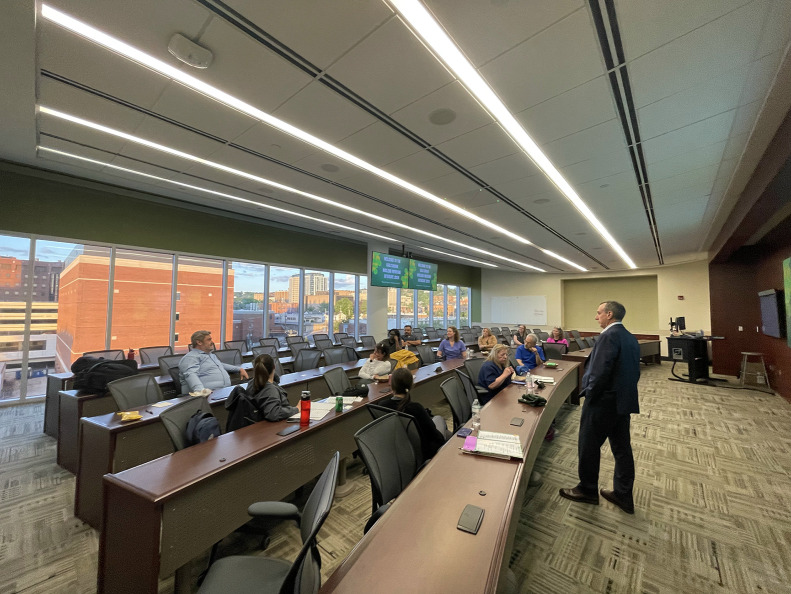
Industry partners engage directly with participants through interactive demonstrations, software training, and discussion.

Industry engagement also fostered networking opportunities, deepened understanding of practical implementation, and prepared participants to return to their institutions with actionable insight (Fig. 6). Most importantly, this collaboration created a model for aligning academic, clinical, and industry partners to accelerate the safe and effective growth of theranostic programs across the country and the world.

### Signature Innovation: The Nuclear Medicine Therapy Escape Room

**FIGURE 7.**
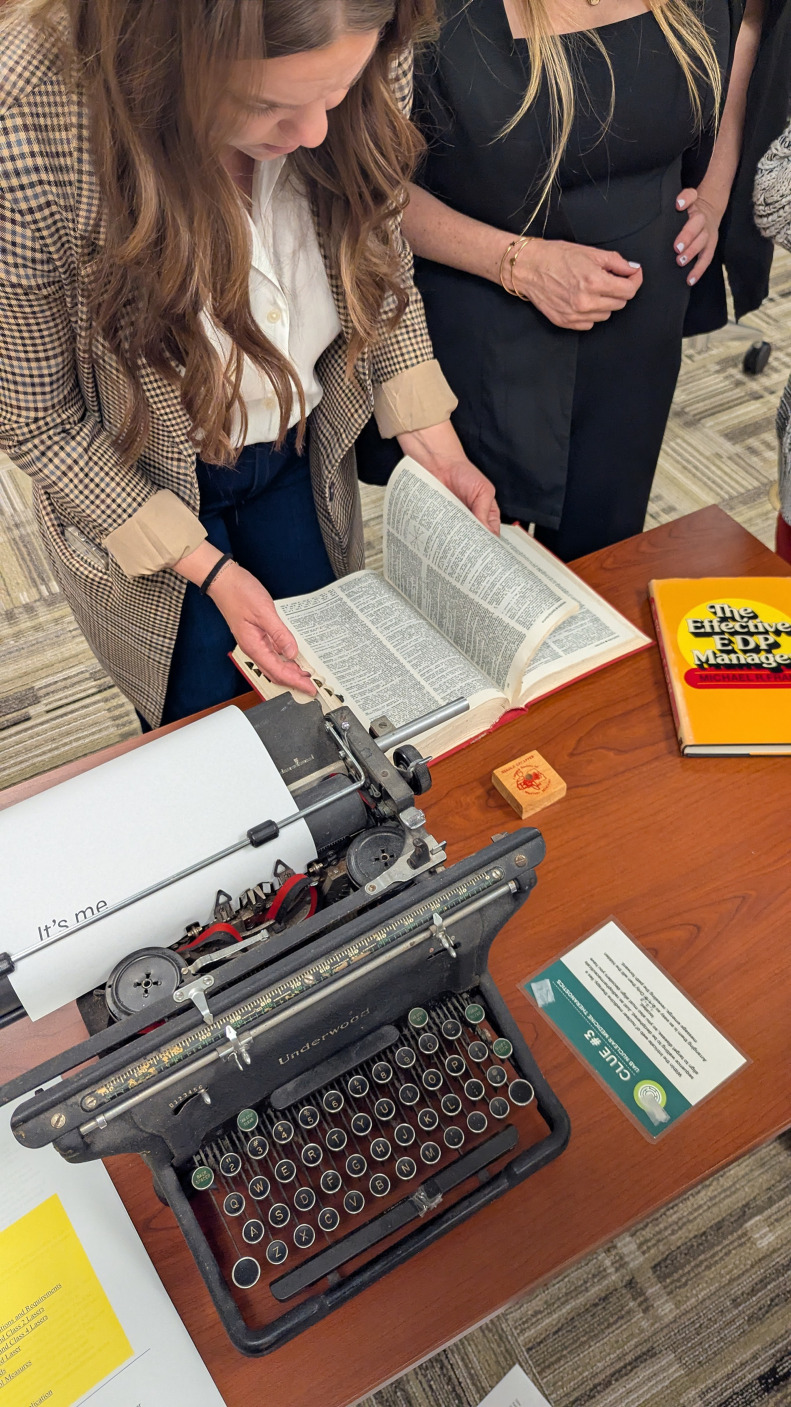
Participants collaborate in the nuclear medicine therapy escape room, a capstone event designed to apply clinical reasoning, radiopharmaceutical selection, and workflow problem-solving in a timed, team-based setting.

The program culminated in the nuclear medicine therapy escape room, a first-of-its-kind capstone event in the field of nuclear medicine therapy. Teams solved case-based puzzles that required clinical reasoning, dose calculation, radiopharmaceutical selection, and workflow problem-solving while under the pressure of the time clock (Fig. 7).

Evidence supports the impact of escape room learning. Reviews and meta-analyses have found that these experiences improve cognitive, psychomotor, affective, and nontechnical skills. Research has shown that escape rooms often outperform traditional methods of enhancing knowledge, teamwork, satisfaction, interprofessional abilities, and reducing anxiety (5,6). Participants consistently described the escape room as a memorable experience. The room was high energy, practical, and applicable to real-world clinical scenarios while also serving as a supercharged, fun activity.

### Program Outcomes and Broader Impact

**FIGURE 8.**
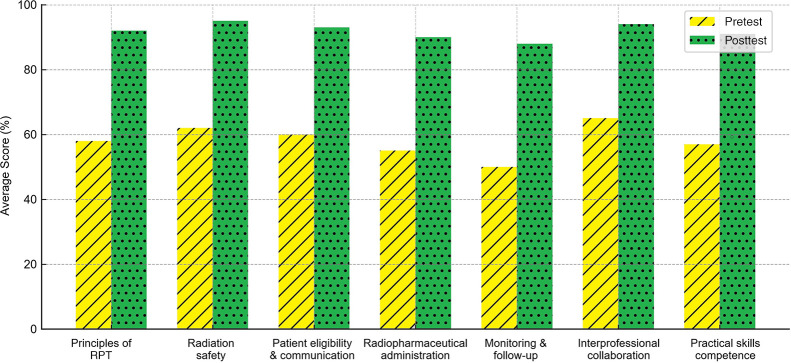
Knowledge gains from UAB/SNMMI Nuclear Medicine Therapy Intensive. This bar chart illustrates how 2024 and 2025 participants advanced from varying levels of baseline knowledge to near-universal mastery across all domains by end of intensive.

This project was reviewed by the UAB Office of the Institutional Review Board and was determined to be *Not Human Subjects Research (Not Research – Training Program Evaluation)*. As such, the project is not subject to Food and Drug Administration regulations for human research. The effectiveness of the intensive was demonstrated through preassessment and postassessment outcomes from the inaugural 2024 and subsequent 2025 cohorts (Fig. 8). In 2024, participants showed marked improvement in knowledge of therapy workflows, eligibility assessment, and regulatory compliance. The 2025 cohort further highlighted the program’s impact. Preassessment data revealed gaps in advanced areas, such as waste contamination control, radiation risk management, and therapy-specific imaging interpretation. By the conclusion of the program, postassessment results showed near-universal mastery, with more than 90%, and in some cases 100%, accuracy, in segmentation, prostate-specific membrane antigen biology, therapy workflows, and interprofessional team roles.

**FIGURE 9.**
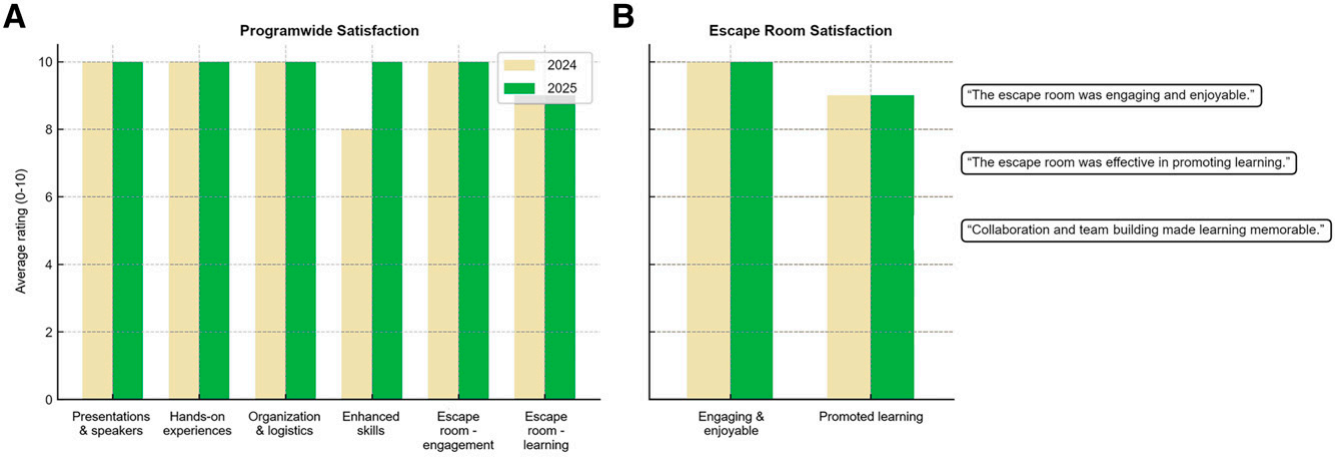
Program satisfaction and escape room satisfaction (2024 vs. 2025). Across both years, participants reported high satisfaction with program quality, organization, and hands-on learning. Escape room activity was consistently rated as engaging and effective in reinforcing learning objectives.

Satisfaction surveys reinforced these findings (Fig. 9). In both years, participants strongly agreed that the program’s presentations, hands-on experiences, and organization enhanced their knowledge and skills, with every respondent indicating that they would recommend the course to colleagues. The capstone Nuclear Medicine Theranostic Escape Room received particularly strong evaluations, with 100% of participants in both years rating it as engaging and enjoyable. In 2024, over 90% reported that it was effective for learning, which rose to a unanimous 100% in 2025.

Participant reflections further highlight the program’s impact. Attendees emphasized both the professional and personal value of the intensive experience. Here are a few quotes:

- “I feel so much better equipped and confident to move forward with theranostics at my facility.” (2024 participant)
- “The presenters took complicated topics and made them very understandable.” (2025 participant)
- “I loved the collaboration and new network of nuclear medicine professionals I can call on when I need advice in the future.” (2024 participant)
- “The escape room and simulations were engaging, effective, and fun ways to learn.” (2025 participant)

Together these outcomes underscore the program’s success in bridging critical knowledge gaps and in teaching participants how to apply these complex concepts with confidence in their workplace. By combining didactic instruction, simulation, and innovative active learning, the Nuclear Medicine Therapy Intensive prepares technologists and advanced practice providers to deliver radiopharmaceutical therapies safely, effectively, and with a patient-centered focus.

### Participant Impact and Professional Growth

**FIGURE 10.**
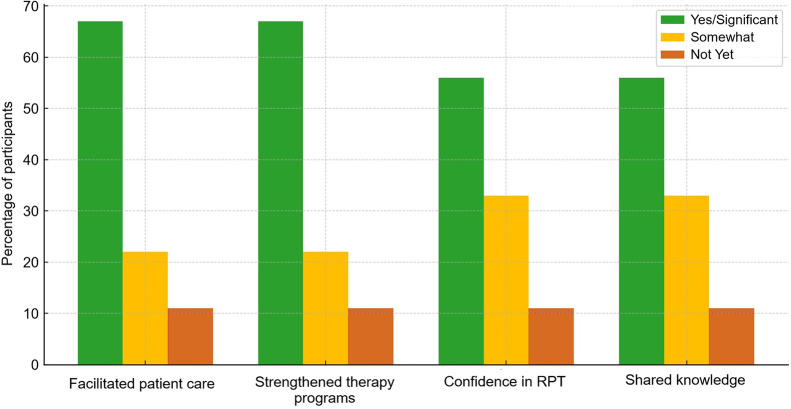
Cohorts who participated in impact survey in 2024 and 2025.

**FIGURE 11.**
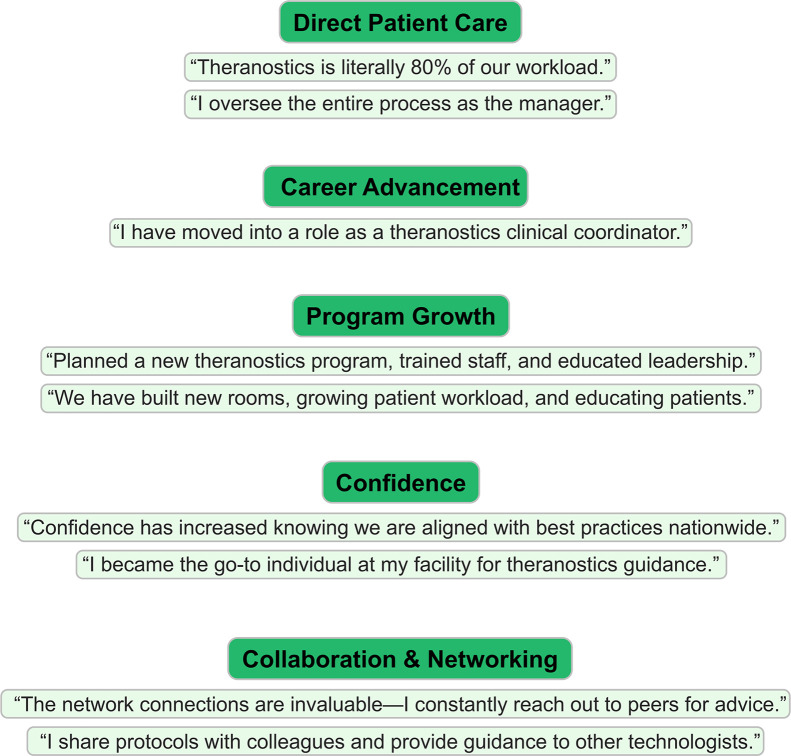
Quotes from impact survey respondents.

A follow-up impact survey was created and sent to both cohorts of participants after several months had passed since their participation in the program (Figs. 10 and 11). The surveys from both the 2024 and 2025 cohorts show that the program is doing exactly what we hoped it would: moving knowledge into practice. More than two thirds of participants reported that they are now directly facilitating patient care in theranostics at their institutions, while others shared that they are actively preparing to do so. Just as importantly, two thirds said they had helped to grow or strengthen therapy programs, whether that meant launching new services, expanding access, developing protocols, or training colleagues.

Confidence was another clear outcome. Almost 90% of participants reported that their confidence in performing or assisting with therapies increased, with over half describing it as a significant boost. Nearly 90% of participants have already shared their knowledge with others, through formal training, workshops, or informal mentoring and case discussions with their teams, showing that their learning was not siloed.

The comments speak volumes about the experience. One participant wrote, “Theranostics is literally 80% of our workload now,” while another shared that they were promoted into a theranostics clinic coordinator role after participating in the program. Several participants described building or expanding programs: “We planned and launched new theranostics services, educated hospital leadership, and trained staff to support patient care.” Others pointed to increased confidence: “I became the go-to individual at my facility for theranostics guidance.” A recurring theme was the network built: “The connections are invaluable…I constantly reach out to peers for advice.”

Taken together, these results make it clear that the intensive is more than a weeklong course. It is a catalyst for patient care, program growth, and workforce development. By building skills, instilling confidence, and creating a professional community, this program is helping NMTs and NMAAs expand access to therapies and strengthen the future of our field.

## HOW TO APPLY

Applications are open to board-certified NMTs and NMAAs who are currently in active practice. Candidates should be prepared to participate fully in all program components. Table 2 summarizes application submission requirements.

**TABLE 2.** Application Submission Summary

Submission requirement	Description
Letter of recommendation	A letter from either your employer or SNMMI/SNMMI-TS leadership is required. Applications will not be reviewed without this document.
Statement of purpose	A well-structured essay describing your reasons for applying to the Nuclear Medicine Therapy Intensive program. The statement should explain how the program aligns with your career goals, outline the specific skills or knowledge you wish to gain, and describe how you intend to apply these learnings in your current role.
Video submission	A short video (mp4 format, not exceeding 3 min) explaining why you would be an excellent candidate. The video should clearly convey your enthusiasm, communication skills, and unique perspective on the field. You may discuss your professional journey, your passion for nuclear medicine, and the ways in which the program would contribute to your growth.
Specific experience in nuclear medicine therapies	A summary of your direct experience with nuclear medicine therapies, including the types of therapies performed, your role in these procedures, and any notable outcomes or challenges encountered.

TS = Technologist Section.

Typically, applications open on December 1 of each year and close on December 31. Notification of acceptance is delivered by February 1. Enrollment is capped to preserve small-group interaction and personalized mentorship. Because of high demand and limited capacity, early application is strongly encouraged. Once all applications are received, they are reviewed and evaluated by the admission committee. Those who are accepted, placed on a waiting list, and not accepted are then notified. Applicants who were not accepted are strongly encouraged to reapply the following year.

## CONCLUSION

The weeklong UAB/SNMMI Nuclear Medicine Therapy Intensive demonstrates that advanced clinical education can be rigorous, engaging, practice-ready, and fun. Currently, the intensive is offered only annually in the spring; however, the hope is to encourage other sites to incorporate this intensive as well. Its integration of foundational science, applied skills, and immersive experiential learning, culminating in the innovative nuclear medicine therapy escape room, serves as a strong model for preparing the next generation of leaders in RPT.
